# Burdens of Tracheal, Bronchus, and Lung Cancer From 1990 to 2021 in China Compared to the Global Projection of 2036: Findings From the 2021 Global Burden of Disease Study

**DOI:** 10.1111/1759-7714.15524

**Published:** 2025-01-22

**Authors:** Yuxing Chen, Qingpeng Zeng, Muyu Li, Jiahui Jin, Jun Zhao

**Affiliations:** ^1^ Department of Thoracic Surgery National Cancer Center/National Clinical Research Center for Cancer/Cancer Hospital, Chinese Academy of Medical Sciences and Peking Union Medical College Beijing China

**Keywords:** age‐standardized rates, China, Global Burden of Disease, prediction models, TBL cancers

## Abstract

**Background:**

Tracheal, bronchial, and lung cancers (TBL cancers) pose a significant global health challenge, with rising incidence and mortality rates, particularly in China. Studies from the Global Burden of Disease (GBD), 2021, can guide screening and prevention strategies for TBL cancer. This study aims to provide a comprehensive analysis of the burden of TBL cancers in China compared to global data.

**Methods:**

We conducted an analysis of incidence, prevalence, mortality, and disability‐adjusted life years (DALYs) from 1990 to 2021. We also performed Joinpoint regression analysis and Bayesian age‐period‐cohort (BAPC) modeling to project future trends.

**Results:**

From 1990 to 2021, there was a substantial increase in TBL cancer indicators for all sexes in China, with the most significant rise observed in females. The female population showed alarming increases in age‐standardized incidence rate (ASIR) and age‐standardized prevalence rate (ASPR). While global efforts have managed to stabilize these rates, China's figures remain high, suggesting the impact of persistent risk factors such as smoking and air pollution, coupled with an aging population. Furthermore, we utilized the projection model in China to estimate that these indicators of TBL cancers in females will likely follow continuous and rapid upward trends, while the burden of TBL cancers among males is expected to have a steady trend.

**Conclusion:**

Although global efforts have been effective in reducing the burden of TBL cancers over the past three decades, there still remains strong regional and gender heterogeneity. TBL cancers need more screening strategies and medical attention in China and in the female population.

## Graphical abstract

TBL cancers are one of the most malignant cancers, with more than 2.3 million new cases worldwide and 0.93 million cases in China in 2021. In the current review, we present the trend of TBL cancer burden in 2021 and projections for 15 years later in China compared with global levels and analyze the data by gender. The graphical abstract shows the statistics worldwide and in China from 1990 to 2021 and projections to 2036.

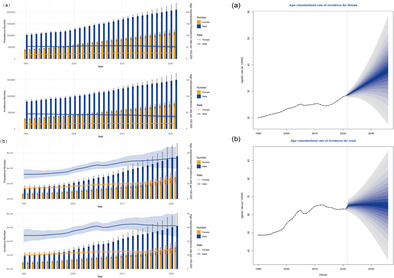

## 1 Introduction

Respiratory system cancer has long been a global health issue, with tracheal, bronchial, and lung cancers (TBL cancers) ranking second in incidence and first in mortality worldwide [1]. Despite extensive research into its epidemiology, the global burden of TBL cancers continued to increase, exacerbated by factors such as smoking prevalence, aging population, and environmental pollutants [2, 3, 4, 5, 6, 7]. While studies have delineated the overall global impact of TBL cancers, regional disparities in incidence, prevalence, and mortality rates are evident [8, 9, 10, 11], underscoring the need for localized data to inform healthcare strategies.

China, with its vast population exceeding 1.4 billion, stands as a critical region for understanding the burden of TBL cancers. The country's demographic and economic transitions have led to unique epidemiological profiles that are essential to comprehend within the broader context of global health. Moreover, China's rapid industrialization and urbanization have introduced additional risk factors that may influence the pattern of TBL cancers. Furthermore, the aging population in China presents additional challenges. As age is a risk factor for TBL cancers [6], the increasing proportion of elderly individuals is likely to augment the disease burden substantially. This demographic shift necessitates a reevaluation of the current healthcare infrastructure and resource allocation to manage the anticipated rise in TBL cancer cases.

Previous studies have confirmed that TBL cancers, with lung cancer being the most prominent, lead in both incidence and mortality among all cancers in China. Notably, there is a rapid escalation in these rates, with a particularly pronounced increase among females [12, 13, 14, 15]. To our knowledge, the Global Burden of Disease (GBD) study represents a continuous and comprehensive updated epidemiological database, which quantifies health loss across the globe due to diseases, injuries, and risk factors. However, with the release of the GBD 2021 database, there exists a paucity of comprehensive studies comparing the latest disease burden of TBL cancers in China with global data. Such comparative analyses are crucial for understanding the unique aspects of TBL cancers' impact in China and for the development of targeted interventions to mitigate this burden.

This study aims to bridge this knowledge gap by providing an updated, comprehensive analysis of the TBL cancer burden within China in comparison to global figures. By doing so, it seeks to highlight the specific needs and challenges in tackling TBL cancers in the context of an aging and populous nation, offering insights that may be applicable to other regions with similar demographic characteristics.

## 2 Methods

### 2.1 Data Sources

Our research conducted a secondary analysis of data from the GBD 2021 study, which utilized available data to provide an assessment of the disease burden of 369 injuries and diseases by sex and age in 204 countries or territories [16]. We extracted the incidence, prevalence, mortality, and the disability‐adjusted life years (DALYs) of TBL cancers worldwide and in China for both sexes from 1990 to 2021 as well as the crude incidence rate (CIR), crude prevalence rate (CPR), crude mortality rate (CMR), and crude DALY rate (CDR) for each age group, via the online data source Global Health Data Exchange (GHDx) query tool (http://ghdx.healthdata.org/gbd‐results‐tool), which uses the International Classification of Diseases 10 (ICD‐10) with the code [C56.9] for TBL cancers as the standard diagnostic tool to categorize diseases. DALYs combine the number of years lived with disability (YLDs) and life lost (YLLs) due to premature mortality. One DALY means the loss of 1 year of full health, providing a comprehensive metric for comparing the burden of various diseases and injuries [17, 18].

In our study, the population with TBL cancers was stratified into age groups at 5‐year intervals. The aforementioned indicators on TBL cancers were collected for both genders within specified age cohorts (0–4, 5–9, 10–14, 15–19, 20–24, 25–29, 30–34, 35–39, 40–44, 45–49, 50–54, 55–59, 60–64, 65–69, 70–74, 75–79, 80–84, 85–89, 90–94, and > 95 years) both worldwide and in China.

### 2.2 Statistical Analysis

To ensure data quality and consistency, we implemented a comprehensive analysis of age‐standardized incidence rates (ASIRs), age‐standardized prevalence rates (ASPRs), age‐standardized mortality rates (ASMRs), and age‐standardized disability‐adjusted life years (ASDRs) for TBL cancers to eliminate the influence of differing age structures between China and the global population. The age‐standardized rate (ASR) represents a summary measure of the rate, which is calculated as a weighted mean of the age‐specific rates per 100 000 population, with the corresponding age groups defined by the World Health Organization (WHO).

For detecting significant changes in temporal trends, Joinpoint regression analysis was performed using the Joinpoint software (version 5.0.2), which identifies points where a statistically significant change in the trend occurs. We calculated the average annual percentage change (AAPC) and the corresponding 95% confidence interval (95% CI) to determine the burden trend of the disease. Log‐transformed age‐standardized indicators can be modeled using a regression framework, expressed as ln(*y*) = *α* + *βx* + *ε*, where *y* represents the age‐standardized indicator in question and *x* denotes the year. The AAPC is calculated as 100 × (exp(*β*) − 1), with the 95% CI derivable from the model. If the 95% CI of the AAPC estimate is greater than zero, there is an increasing trend in the age‐standardized indicator; if it is less than zero, there is a decreasing trend; and if it includes zero, a stable trend is observed.

To elucidate the demographic factors influencing the epidemiology of TBL cancers, we employed decomposition analysis to visually demonstrate the role of the three factors driving changes in ASIR, ASPR, ASMR, and ASDR between 1990 and 2021, by gender (i.e., age, population, and epidemiology) [19]. The methodology entailed assessing the contribution of each factor in isolation, with the other two factors remaining fixed [20].

The Bayesian age‐period‐cohort (BAPC) model was utilized to further predict the ASIR, ASPR, ASMR, and ASDR of TBL cancers from 2022 to 2036 [21]. The uncertainty of estimates was quantified using 95% uncertainty intervals (UI), representing the range within which the true values are expected to lie with 95% probability. The BAPC model relies on integrated nested Laplace approximations (INLA) to estimate marginal posterior distributions, which helps circumvent some of the mixing and convergence issues associated with traditional Markov chain Monte Carlo (MCMC) sampling Bayesian methods. The Nordpred package in R software was used to analyze the ASIR and ASDR of TBL cancers from 1990 to 2036, and we used the BAPC and INLA packages for analysis to verify the reliability of the predicted results.

The software R (version 4.3.0) was employed for all other statistical computations and modeling procedures. Throughout the analysis, a *p*‐value of less than 0.05 was considered statistically significant [22].

## 3 Results

### 3.1 Incidence of TBL Cancers Worldwide and in China

**FIGURE 1:**
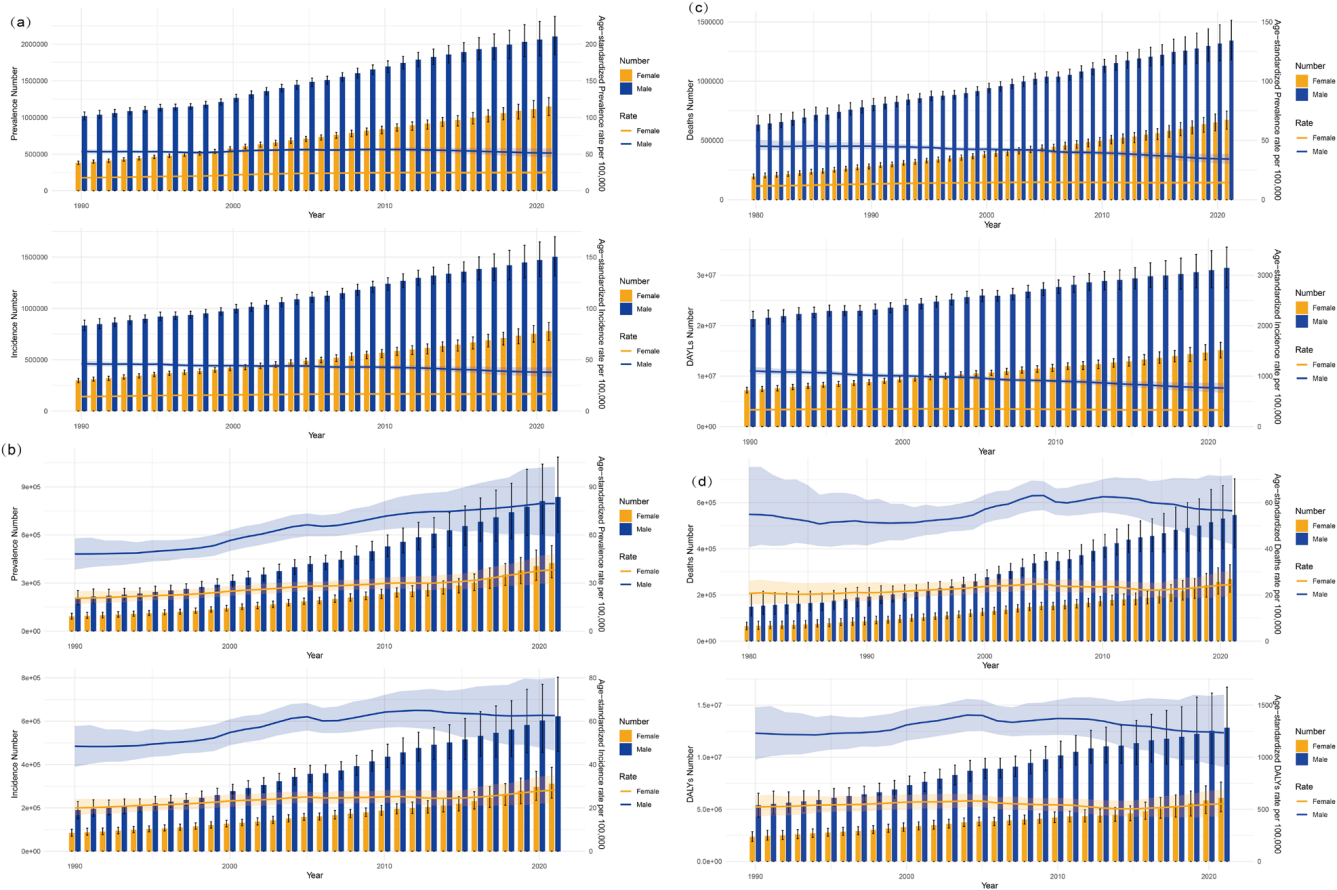
(a) Incidence and prevalence of TBL cancers worldwide from 1990 to 2021. (b) Incidence and prevalence of TBL cancers in China from 1990 to 2021. (c) Mortality and DALYs of TBL cancers worldwide from 1990 to 2021. (d) Mortality and DALYs of TBL cancers in China from 1990 to 2021.

The overall data are presented in Table 1 and Figure 1. Worldwide, for incidence, the number of TBL cancers demonstrated significantly increasing trends from 1 132 064 (95% CI: 1 075 371–1 186 163) in 1990 to 2 280 688 (95% CI: 2 063 252–2 509 740) in 2021. In China, the number of newly diagnosed cases of TBL cancers was 274 752 (95% CI: 234 741–315 112) in 1990 and 934 704 (95% CI: 750 040–1 136 938) in 2021. The change in numbers of incidences between 1990 and 2021 represents cumulative increases of 101.46% and 240.20%, worldwide and in China, respectively. However, the ASIR of TBL cancers demonstrated a divergent trend. Globally, ASIR exhibited a marginal decline, shifting from 28.54 (95% CI: 27.06–29.91) per 100 000 population in 1990 to 26.43 (95% CI: 23.9–29.07) in 2021. Conversely, a significant increase was observed in China, with the ASIR rising from 33.11 (95% CI: 28.47–37.79) to 44.1 (95% CI: 35.45–53.35). Meanwhile, the AAPC of the ASIR of TBL cancers worldwide decreased by −0.27% (95% CI: −0.36–0.17, *p* < 0.01) from 1990 to 2021, and in China, it increased by 0.94 (95% CI: 0.75–1.13, *p* < 0.01).

**TABLE 1:** Incidence, prevalence, mortality, and DALYs worldwide and in China.

Location	Measure	Sex	1990		2021		
			All‐age cases	Age‐standardized rates per 100 000 people	All‐age cases	Age‐standardized rates per 100 000 people	1990–2021 AAPC
			*n* (95% CI)	*n* (95% CI)	*n* (95% CI)	*n* (95% CI)	*n* (95% CI)
China	Incidence	Both	274 752 (234 741–315 112)	33.11 (28.47–37.79)	934 704 (750 040–1 136 938)	44.01 (35.45–53.35)	0.94 (0.75–1.13)
		Female	84 507 (69 112–101 782)	19.97 (16.45–23.98)	311 944 (245 717–387 211)	28.16 (22.22–34.90)	1.18 (1.01–1.34)
		Male	190 245 (150 986–229 441)	48.46 (38.94–57.75)	622 760 (460 105–803 234)	62.63 (46.50–79.90)	0.85 (0.66–1.05)
	Prevalence	Both	301 999 (257 252–347 798)	33.74 (28.90–38.65)	1 262 275 (1 005 551–1 545 341)	57.95 (46.20–70.78)	1.81 (1.60–2.02)
		Female	92 191 (74 676–111 671)	20.46 (16.68–24.66)	425 701 (333 642–533 493)	38.34 (30.09–48.08)	2.10 (1.94–2.25)
		Male	209 808 (165 996–254 011)	48.13 (38.43–57.83)	836 574 (616 941–1 085 694)	79.57 (58.95–102.56)	1.63 (1.43–1.84)
	Deaths	Both	278 226 (238 194–318 827)	34.74 (29.96–39.52)	814 364 (652 636–987 795)	38.98 (31.40–47.06)	0.38 (0.15–0.61)
		Female	85 817 (70 530–103 029)	20.91 (17.36–25.18)	268 402 (211 859–331 292)	24.42 (19.30–30.11)	0.53 (0.34–0.72)
		Male	192 409 (152 808–231 623)	51.45 (41.44–61.14)	545 962 (403 556–702 866)	56.45 (41.89–71.82)	0.34 (0.11–0.56)
	DALYs	Both	7 62 374 (6 610 051–8 947 038)	863.54 (738.86–991.39)	18 920 203 (15 100 681–23 111 519)	878.24 (703.53–1068.71)	0.06 (−0.12–0.24)
		Female	2 358 325 (1 915 033–2 852 517)	522.43 (426.02–628.98)	6 088 330 (4 755 094–7 606 766)	553.00 (431.58–690.27)	0.18 (0.01–0.36)
		Male	5 404 050 (4 264 070–6 544 173)	1230.74 (979.34–1478.69)	12 831 873 (9 352 167–16 730 756)	1235.03 (905.79–1600.15)	0.01 (−0.17–0.20)

Global	Incidence	Both	1 132 064 (1 075 371–1 186 163)	28.54 (27.06–29.91)	2 280 688 (2063252–2 509 740)	26.43 (23.9–29.07)	−0.27 (−0.36–0.17)
		Female	299 151 (276 653–319 390)	14.05 (12.98–15)	778 708 (689 762–862 190)	16.8 (14.9–18.6)	0.58 (0.51–0.65)
		Male	832 913 (785 260–886 177)	46.16 (43.58–49.03)	1 501 980 (1317171–1 697 521)	37.85 (33.3–42.67)	−0.66 (−0.76–0.56)
	Prevalence	Both	1 399 192 (1 335 754–1 458 319)	34.25 (32.66–35.69)	3 253 729 (2 947 831–3 558 836)	37.28 (33.76–40.77)	0.27 (0.18–0.35)
		Female	381 144 (356712‐403 802)	17.62 (16.47–18.66)	1 150 188 (1026791–1 268 775)	24.95 (22.28–27.52)	1.12 (1.02–1.23)
		Male	1 018 048 (965387–1 075 042)	53.59 (50.9–56.49)	2 103 542 (1 856 371–2 376 979)	51.5 (45.62–57.98)	−0.14 (−0.25–0.04)
	Deaths	Both	1 080 128 (1 023 327–1 135 557)	27.58 (26.09–28.99)	2 016 547 (1 820 498–2 218 372)	23.5 (21.22–25.85)	−0.54 (−0.66–0.42)
		Female	281 306 (259 310–301 716)	13.31 (12.24–14.27)	674 006 (595 386–747 508)	14.51 (12.83–16.1)	0.27 (0.20–0.33)
		Male	798 821 (750 843–853 582)	45.26 (42.63–48.28)	1 342 541 (1 180 489–1 513 563)	34.32 (30.26–38.57)	−0.92 (−1.02–0.81)
	DALYs	Both	28 459 836 (26 973 713–29 909 115)	690.86 (654.39–725.97)	46 536 272 (41 903 412–51 205 051)	533 (480.13–586.36)	−0.86 (−0.98–0.75)
		Female	7 175 101 (6 643 722–7 743 271)	330.93 (306.32–356.76)	15 096 643 (13 599 782–16 694 421)	328.78 (296.62–363.63)	−0.03 (−0.10–0.03)
		Male	21 284 735 (19 931 198–22 827 664)	1102.45 (1034.44–1180.38)	31 439 630 (27 496 302–35 558 586)	763.74 (668.59–862.68)	−1.21 (−1.31–1.10)

### 3.2 Prevalence of TBL Cancers Worldwide and in China

Globally, the number of TBL cancers increased from 1 399 192 (95% CI: 1 335 754–1 458 319) in 1990 to 3 253 729 (95% CI: 2 947 831–3 558 836) in 2021. The ASPR increased from 34.25 (95% CI: 32.66–35.69) to 37.28 (95% CI: 33.76–40.77). However, in China, it increased from 301 999 (95% CI: 257 252–347 798) in 1990 to 1 262 275 (95% CI: 1 005 551–1 545 341) in 2019 and changed by 317.97%. The ASPR increased from 33.74 (95% CI: 28.90–38.65) to 57.95 (95% CI: 46.20–70.78). The AAPC of the ASPR increased by 0.27% (95% CI: 0.18–0.35, *p* < 0.01) from 1990 to 2021 worldwide and by 1.81% (95% CI: 1.60–2.02, *p* < 0.01) in China.

### 3.3 Deaths due to TBL Cancers Worldwide and in China

Worldwide, TBL cancer caused 1 080 128 (95% CI: 1 023 327–1 135 557) death cases in 1990 and 2 016 547 (95% CI: 1 820 498–2 218 372) death cases in 2021, showing an 86.70% increase in the observed period. In China, the number of deaths attributable to TBL cancers demonstrated the same trend, which increased from 278 226 (95% CI: 238 194–318 827) to 814 364 (95% CI: 652 636–987 795), representing a 192.70% increase compared to that in 1990. Besides, the ASMR demonstrated a different trend. Globally, the AMSR of TBL cancers decreased from 27.58 (95% CI: 26.09–28.99) per 100 000 population in 1990 to 23.5 (95% CI: 21.22–25.85) in 2021. In China, the ASMR increased from 34.74 (95% CI: 29.96–39.52) per 100 000 population in 1990 to 38.98 (95% CI: 31.40–47.06) in 2021. The AAPCs of the mortality rate were − 0.54 (95% CI: −0.66–0.42, *p* < 0.01) worldwide and 0.38 (95% CI: 0.15–0.61, *p* < 0.01) in China.

### 3.4 DALYs of TBL Cancers Worldwide and in China

Globally, the DALYs caused by TBL cancer were 46 536 272 (95% CI: 41 903 412–51 205 051) in 2021, showing a 63.52% increase compared to that in 1990. In China, the DALYs of TBL cancer were 18 920 203 (95% CI: 15 100 681–23 111 519), showing a 143.74% increase compared to that in 1990. For age‐standardized DALYs, it dropped significantly from 690.86 (95% CI: 654.39–725.97) to 533 (95% CI: 480.13–586.36) during this period worldwide and showed a relatively smoother increase from 863.54 (95% CI: 738.86–991.39) to 878.24 (95% CI: 703.53–1068.71). The AAPCs of age‐standardized DALYs were −0.86 (95% CI: −0.98–0.75, *p* < 0.01) and 0.06 (95% CI: −0.12–0.24, *p* = 0.50), respectively.

### 3.5 Variation of TBL Cancer Burden in the Two Sexes

In the observed period, significant disparities exist between males and females in ASIR, ASPR, ASMR, and age‐standard DALYs of TBL cancers, both globally and within China (Figure 1). For females, the trends of ASIR, ASPR, and ASMR showed a significant increase from 1990 to 2021 both worldwide and in China. In particular, the upward trend in China is markedly higher than the global average. Besides, DALYs have remained relatively stable globally while exhibiting a slight increase in China. For males, globally, the ASIR, ASPR, ASMR, and age‐standard DALYs for TBL cancers in males have begun to show a declining trend, whereas in China, they remain on an upward trend.

### 3.6 Variation of TBL Cancer Burden in Different Age Groups

The age distribution of the burden of TBL cancers demonstrated no significant difference between China and global trends in 2021. Figure S1 shows the number of incidence, prevalence, deaths, and DALYs of TBL cancers in different age groups of males and females worldwide and in China in 2021. The peak incidence and death numbers of TBL cancer were observed in the age group of 70–74 years, irrespective of gender and geographic location. The peak prevalence and DALYs for TBL cancers were observed in the 65–69 years age cohort. Besides, in nearly all age groups, the burden of TBL cancers in males was significantly higher than that in females, in terms of both numbers and rates.

### 3.7 Joinpoint Regression Analysis of TBL Cancer Burden

**FIGURE 2:**
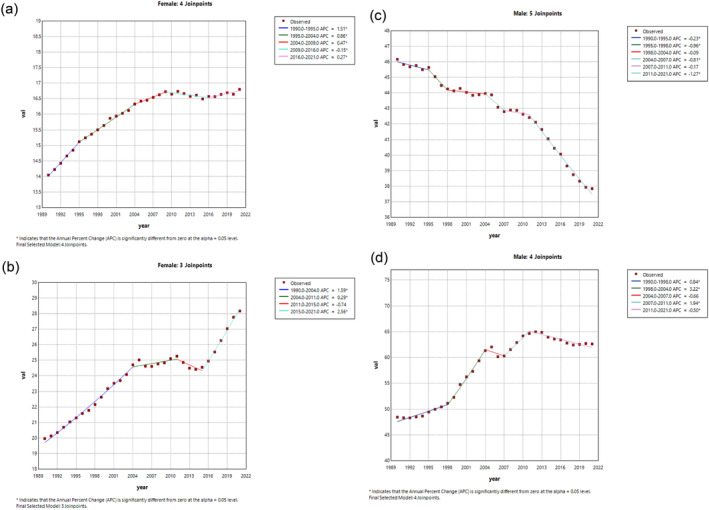
(a) Joinpoint regression analysis of TBL cancers in ASIR for females worldwide. (b) Joinpoint regression analysis of TBL cancers in ASIR for females in China. (c) Joinpoint regression analysis of TBL cancers in ASIR for males worldwide. (d) Joinpoint regression analysis of TBL cancers in ASIR for males in China.

The Joinpoint regression analyses of ASIR in females and males for TBL cancers in China and worldwide from 1990 to 2021 are demonstrated in Figure 2. The results of the ASPR, ASMR, and DALY rates by different sex groups in China and worldwide are demonstrated in Figure S2. For females, we found that the trend of ASIR TBL cancers significantly increased from 1990 to 2009 in both China and worldwide. After 2009, the global increase in the ASIR of TBL cancer began to decelerate, whereas in China, a rapid ascent was observed once again starting in 2015. For males, a declining trend in the ASIR of lung cancer was observed globally from 1990 to 2021, while in China, the decrease commenced in the year 2011.

### 3.8 Decomposition Analysis

A decomposition analysis was conducted to evaluate the changes in incidence, prevalence, mortality, and DALYs across global and Chinese populations to quantify the contributions of aging, population growth, and epidemiological changes to these health metrics. The details of decomposition analysis results are shown in Figure S3 and Table 2. Globally, the increase in incidence, prevalence, mortality, and DALY rates was predominantly driven by population, accounting for 62.96%, 49.00%, 57.82%, and 85.33%, respectively. In China, the trends in these indices were different from the global patterns, with a more pronounced impact from aging, accounting for 63.56%, 42.41%, 64.15%, and 79.81%.

**TABLE 2:** Decomposition analysis of TBL cancer worldwide and in China.

	Sex	Incidence changes			Prevalence changes		
		Age	Population	Epidemiological change	Age	Population	Epidemiological change
Global	Both	122 703.62 (9.41%)	820 707.07 (62.96%)	360 045.53 (27.62%)	748675.48 (45.96%)	798103.00 (49.00%)	82161.26 (5.04%)
	Female	86331.08 (12.72%)	596311.24 (87.83%)	−3680.12 (−0.54%)	222828.72 (32.54%)	253720.33 (37.05%)	208170.91 (30.40%)
	Male	−3521.70 (−0.59%)	286673.69 (47.90%)	315354.36 (52.69%)	547523.40 (57.73%)	543828.22 (57.34%)	−142879.86 (−15.06%)
China	Both	366481.27 (63.56%)	98499.53 (17.08%)	111593.22 (19.35%)	282265.06 (42.41%)	120297.48 (18.08%)	262934.91 (39.51%)
	Female	117831.86 (58.96%)	33536.40 (16.78%)	48489.86 (24.26%)	82202.61 (36.10%)	43766.70 (19.22%)	101740.32 (44.68%)
	Male	257554.09 (66.06%)	64775.97 (16.61%)	67575.81 (17.33%)	186755.90 (41.00%)	80268.90 (17.62%)	188427.83 (41.37%)

	Sex	Mortality changes			DALYs changes		
		Age	Population	Epidemiological change	Age	Population	Epidemiological change
Global	Both	1137671.02 (49.33%)	1333455.26 (57.82%)	−164909.26 (−7.15%)	12802925.67 (78.42%)	13931432.90 (85.33%)	−10408498.31 (−63.75%)
	Female	329710.45 (35.27%)	398181.96 (42.60%)	206817.36 (22.13%)	3461778.56 (47.14%)	4005716.24 (54.54%)	−123375.12 (−1.68%)
	Male	907313.42 (64.16%)	1005712.28 (71.12%)	−498882.08 (−35.28%)	9732546.74 (107.20%)	9925786.95 (109.33%)	−10579233.28 (−116.52%)
China	Both	540126.24 (64.15%)	182261.80 (21.65%)	119586.64 (14.20%)	8180678.30 (79.81%)	2348390.03 (22.91%)	−278381.91 (−2.72%)
	Female	185536.43 (62.73%)	62109.42 (21.00%)	48119.18 (16.27%)	2542559.32 (74.20%)	769980.50 (22.47%)	113860.18 (3.32%)
	Male	395848.86 (62.44%)	129690.42 (20.46%)	108379.74 (17.10%)	5734678.29 (81.98%)	1558452.59 (22.28%)	−297879.04 (−4.26%)

### 3.9 Predictions of TBL Cancer From 2022 to 2036

**FIGURE 3:**
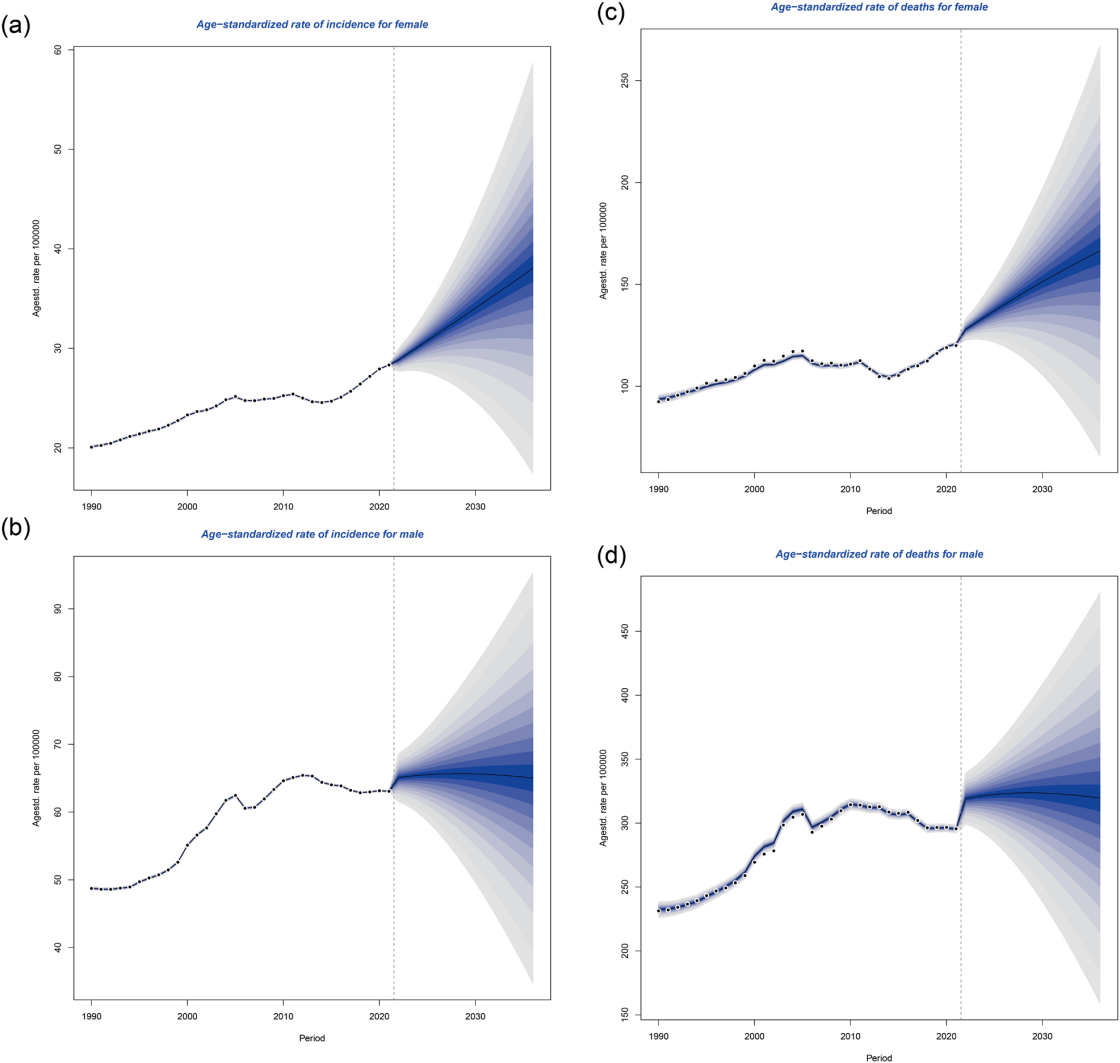
(a) Prediction of TBL cancer incidence from 1990 to 2036 for females in China. (b) Prediction of TBL cancer incidence from 1990 to 2036 for males in China. (c) Prediction of TBL cancer mortality from 1990 to 2036 for females in China. (d) Prediction of TBL cancer mortality from 1990 to 2036 for males in China.

We predicted the ASIR and ASMR of TBL cancer of different sexes in China from 2021 to 2036 by using the BAPC models in China. The ASIR and ASMR in females will experience a rapid and continuous increase from 2022 to 2036. However, in males, the ASIR and ASMR of TBL cancers will cease to increase and tend toward stabilization in the future (Figure 3).

## 4 Discussion

TBL cancers have consistently been a health concern of global significance, characterized by the highest incidence and mortality rates across various regions. Several studies utilizing the GBD database have previously been conducted on TBL cancers. In 2013, TBL cancer ranked first in incidence, with an estimated 1.8 million new cases and 1.6 million deaths. Besides, TBL cancers were responsible for 34.7 million DALYs, with 38% occurring in developed countries and 62% in developing countries [23]. In 2017, there were 2.2 million cases of TBL cancer, 1.9 million deaths, and 40.9 million DALYs [8, 24, 25]. In 2019, there were 2.3 million new cases of TBL cancer, 2.0 million deaths, and 45.8 million DALYs [11].

In our research, we analyzed the results of the GBD 2021 study to determine the burden of TBL cancers worldwide and in China, assess the differential burden by gender, explore its potential causes, and project future trends. In 2021, as previously mentioned, there were 2.3 million cases of TBL cancer, 2.0 million deaths, and 46.5 million DALYs. Notably, in comparison to global averages, the incidence, prevalence, mortality, and DALY rates per 100 000 population of TBL cancers in China are significantly higher. Besides, all of the indicators decreased in males between 1990 and 2021 worldwide, but they had a marked increase in females, except for the DALYs rate. However, in China, all of the indicators increased in both females and males, with the female population exhibiting the most rapid and substantial increasing trend. The observed disparity might be attributable to the following reasons. First, as we all know, smoking is considered the leading risk factor for TBL cancers. As the Framework Convention on Tobacco Control (FCTC) started in 2005 and expanded to 182 countries in 2021 to reduce taxing tobacco and advertising of tobacco products, the burden of TBL cancers declined worldwide due to tobacco control efforts carried out globally [26, 27, 28, 29]. However, tobacco use by China's adult population still remains higher than that in many other countries [30]. Although in China, where smoking by women remained uncommon, indoor air pollution from cooking and heating played a major role in TBL cancer incidence [31]. Apart from this, China has undergone extensive industrialization and urbanization over the past several decades, which has exacerbated environmental pollution [32]. Recent studies have demonstrated that 1.41 billion individuals (99% of the population) suffer from unsafe PM2.5 levels (over 5 μg/m^3^), and 0.765 billion (53%) suffer from hazardous concentrations (over 35 μg/m^3^) [33], which confirmed that the level of PM2.5 has a significant positive association with TBL cancer mortality [34, 35]. On the one hand, due to the pandemic of COVID‐19 and the widespread adoption of low‐dose computed tomography (LDCT), the detection rate of early‐stage lung cancer has increased significantly [36, 37]. Moreover, our findings also indicate that population aging plays a significant role in the increased burden of TBL cancers. It is well‐known that cancer is an age‐associated disease. Due to the pronatalist policies in China during the last century, coupled with the recent decline in marriage and fertility rates, the proportion of the elderly population in China is continuously increasing, which might lead to the increase of the TBL cancer burden.

Besides, although the current burden of TBL cancer is higher in males, the increasing trend in disease burden among female patients is substantial and warrants attention. This phenomenon could be partly explained by several reasons as discussed below. First, females are more sensitive to specific carcinogens in tobacco than males (especially lung adenocarcinoma), which might be due to their sensitivity to gene mutations, like p53 and K‐RAS, as well as the interaction between estrogen and tobacco carcinogens [38]. Besides, epidemiological models indicate that the increase in smoking prevalence among females emerged 20 to 30 years later than that observed in males [39]. In China, effective measures have been implemented to reduce the burden of TBL cancers to control cigarette prevalence and air pollution, resulting in the decrease of tobacco smoking prevalence and PM 2.5 concentrations [40, 41]. However, there are still several questions, like the increase of e‐cigarette use, cooking fumes, polycyclic aromatic hydrocarbon (PAH) exposure, and so on [40, 42]. It is still important for us to realize the great potential of TBL cancer management, and more effective measures are needed to reduce the burden of TBL cancers in China.

In our BAPC prediction models, we have projected the changes in the incidence and mortality rates of TBL cancers in China over the next 15 years. While the incidence and mortality rates in men have begun to stabilize and show a slight decline, the rates in women continue to rise. The decline in disease burden among men may be attributed to the control of smoking, changes in age structure, and improvements in treatment levels. The increase in disease burden among women may be due to the fact that lung cancer incidence in females is more often related to genetic susceptibility rather than smoking, with the EGFR mutation being the most common in East Asian females [43, 44]. Under conditions of environmental pollution, this susceptibility may be further amplified. However, the BAPC model typically uses historical data to make predictions. It may not account for future changes in risk factors, health policies, medical advancements, or other unforeseen events that could affect disease burden. Further studies are imperative upon the release of updated disease burden data to elucidate the evolving epidemiological patterns.

Alleviating the burden of TBL cancers represents a multifaceted challenge that necessitates collaborative efforts from governments, healthcare systems, research institutions, non‐governmental organizations, and the general public. Firstly, exposure to carcinogenic risk factors can be reduced through public health campaigns and the implementation of health policies, such as smoking bans and regulations aimed at decreasing exposure to carcinogens in the environment. It is also significant to enhance disease screening for the elderly and promote education and awareness related to TBL cancers in the aged population. Besides, in recent years, an increasing number of techniques have emerged in the field of TBL cancer treatment, such as targeted therapy, immunotherapy, stereotactic radiotherapy, and radiofrequency ablation therapy, all of which have demonstrated commendable efficacy. It is a great challenge for medical care teams, including thoracic surgeons, medical oncologists, radiation oncologists, pathologists, radiologists, and other relevant departments, to collaborate to provide TBL cancer patients with the most rational and individualized treatment plans to improve their survival time and quality of life. Moreover, the phase III ADAURA analysis demonstrated a clinically significant disease‐free survival (DFS) benefit with adjuvant osimertinib versus placebo in EGFR‐mutated stage IB‐IIIA non‐small‐cell lung cancer (NSCLC) after complete tumor resection [45]. The ALINA trials demonstrated that adjuvant alectinib significantly improved DFS as compared with platinum‐based chemotherapy among patients with resected ALK‐positive NSCLC of stage IB‐IIIA. Despite notable progress achieved in recent clinical studies, there is still an urgent need for high‐quality, multicentric clinical research for research institutions [46].

Moreover, population aging has been a global issue that cannot be ignored, particularly in China. Due to the decline in fertility rates, the proportion of the elderly in China's population structure has increased significantly. TBL cancers are characterized by increasing incidence and mortality rates concomitant with advancing age. Our research revealed that the substantial increase in the disease burden of TBL cancer is predominantly attributed to population aging. TBL cancers in older patients are typically characterized by later‐stage diagnosis and a higher prevalence of internal comorbidities, which contribute to the complexity of treatment modalities. At present, the most widely utilized screening method for TBL cancers is LDCT, which has been confirmed to be effective for the secondary prevention of TBL cancers. The National Lung Screening Trial (NLST) and the Dutch–Belgian Randomized Lung Cancer Screening Trial (NELSON) set the age criteria for lung cancer screening at 55 to 74 years and 50 to 74 years, respectively. Both trials have substantiated the efficacy of LDCT in reducing mortality arising from lung cancer among high‐risk populations, including current and former smokers [47, 48, 49]. Furthermore, The US Preventive Services Task Force has expanded the screening age population to include those aged 50–80 years and individuals who smoke more than 20 packs annually [50]. However, all of the randomized controlled trials have mentioned that screening high‐risk people with LDCT could reduce lung cancer mortality and also cause a range of harms, such as overdiagnosis, unnecessary invasive procedures, and, rarely, radiation‐induced cancer. The formulation of individualized screening protocols is becoming increasingly imperative in response to the persistent morbidity and mortality associated with this disease. These protocols should be tailored to reflect individual risk profiles, incorporating factors such as smoking history, family history of cancer, occupational exposures, age, gender, and socioeconomic status. By stratifying risk and personalizing screening schedules, healthcare systems can optimize the balance between the benefits and potential harms of TBL cancer screening.

However, there are still several limitations in this research. Firstly, although the data we utilized were sourced from the newest GBD 2021 database, it should be noted that our research is a secondary data analysis. In China, the healthcare system, diagnostic criteria, and disease registry mechanisms were not as developed three decades ago as they are today. Global‐level data on the total burden of TBL cancers are presented to depict the overall burden of TBL cancers worldwide. These global data may not serve as direct reference levels for the specific disease burden of individual countries or regions and require further in‐depth analysis based on the particular data of each country or region. The availability and quality of these raw data may impose limitations on the findings of our research. Besides, while this study provides an overview of TBL cancer epidemiology in China, it is important to note the absence of detailed data. With its vast territory of 9.6 million square kilometers, China exhibits substantial regional diversity in terms of environmental and pollution levels, as well as lifestyle among its population. This heterogeneity extends to the epidemiology of TBL cancers, with variations observed between the southern and northern as well as the eastern and western parts of the country [51]. Further research is needed to help formulate the screening policies. Moreover, TBL cancers encompass a variety of pathological types, with lung cancer being classified into two main categories: NSCLC and small cell lung cancer (SCLC). These categories are pivotal for determining the approach to treatment and prognosis. However, because of the limitation of the GBD database, we are not able to conduct a statistical analysis of these cancer subtypes.

## 5 Conclusions

There still remain large disease burdens of TBL cancers in China, which might continue to increase in the following decades. The fact that all of the indices significantly increased in females should not be neglected. Nevertheless, despite the implementation of tobacco control policies in China, the approximately 2‐decade latency period between exposure to cigarette smoke and the development of TBL cancers underscores the importance of regular monitoring of this disease burden and the formulation of appropriate policies in the country. It is also of great significance for us to develop healthy living habits and enhance our awareness of early diagnosis and treatment.

## Author Contributions

Y.C. participated in the conceptualization, literature search, study design, data curation, data analysis, and data interpretation and drafted the original manuscript. J.Z. and Y.C. conceived the study and participated in its design, coordination, data collection, and analysis. Y.C. and Q.Z. participated in the study design and data curation and provided the critical revision. All authors contributed to the article and approved the submitted version.

## Conflicts of Interest

The authors declare no conflicts of interest.

## Supporting information


**Figure S1.** (a) Incidence and prevalence of TBL cancers worldwide by different age groups. (b) Incidence and prevalence of TBL cancers in China by different age groups. (c) Mortality and DALYs of TBL cancers worldwide by different age groups. (d) Mortality and DALYs of TBL cancers in China by different age groups.

**Figure S2.** Joinpoint regression analysis of TBL cancers in ASPR for females (a) and males (b) worldwide, in ASPR for females (c) and males (d) in China, in ASMR for females (e) and males (f) worldwide, in ASMR for females (g) and males (h) in China, in ASDR for females (i) and males (j) worldwide, and in ASDR for females (k) and males (l) in China.

**Figure S3.** Decomposition analysis of ASIR of TBL cancers worldwide (a) and in China (b), ASPR worldwide (c) and in China (d), ASNR worldwide (e) and in China (f), and ASDR worldwide (g) and in China (h).

## Data Availability

In this study, publicly available data sets were analyzed. The name and accession number can be found below: https://ghdx.healthdata.org/gbd‐2021.
