# Supplementary figures and images for: Burdens of Tracheal, Bronchus, and Lung Cancer From 1990 to 2021 in China Compared to the Global Projection of 2036: Findings From the 2021 Global Burden of Disease Study
Source: Thorac Cancer. 2025 Jan 22;16(2):e15524. doi: 10.1111/1759-7714.15524 (PMC11751713; doi:10.1111/1759-7714.15524)

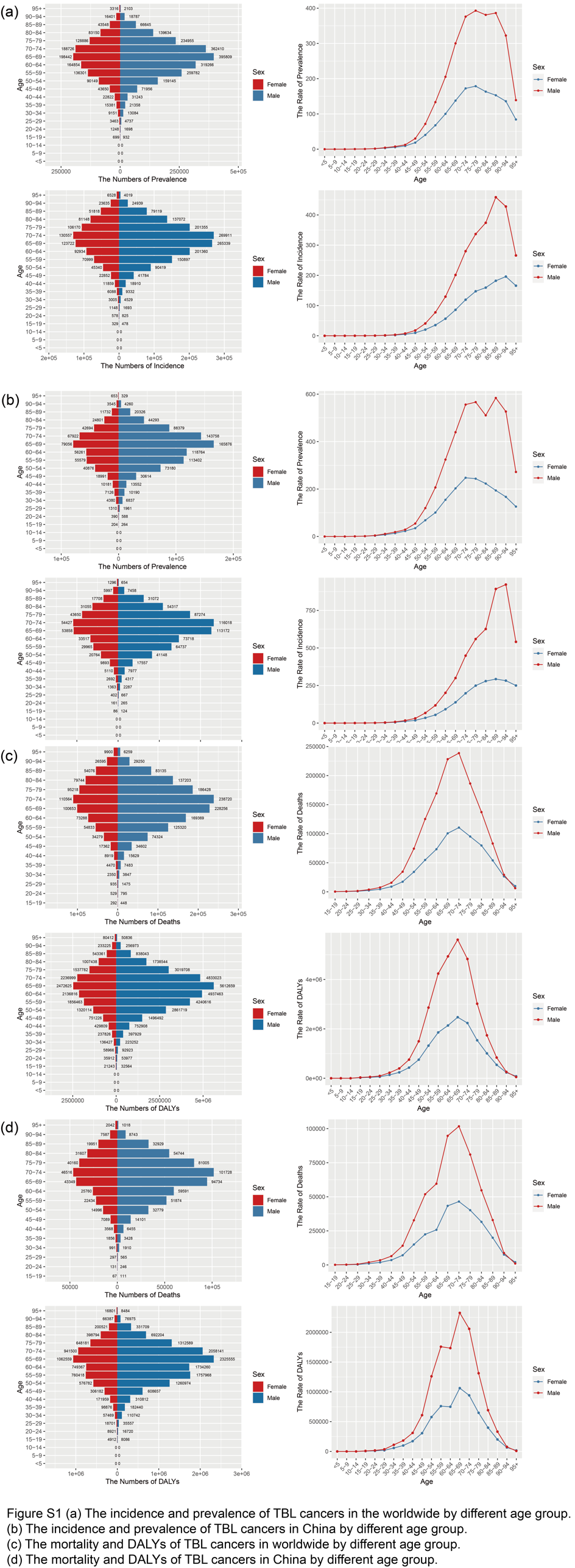

Supplement: Supplementary file 1 — Figure S1. (a) Incidence and prevalence of TBL cancers worldwide by different age groups. (b) Incidence and prevalence of TBL cancers in China by different age groups. (c) Mortality and DALYs of TBL cancers worldwide by different age groups. (d) Mortality and DALYs of TBL cancers in China by different age groups. [file TCA-16-e15524-s001.png]
